# Prognostic value of brain glucose levels in the outcome of patients with spontaneous cerebral hemorrhage

**DOI:** 10.1186/cc9741

**Published:** 2011-03-11

**Authors:** DC Papadopoulos, TK Zafeiridis, M Mpakopoulou, G Paraforos, A Chovas, V Christodoulou, A Komnos

**Affiliations:** 1 General Hospital of Larisa, Greece; 2 University Hospital of Larissa, Greece

## Introduction

Spontaneous cerebral hemorrhage is a major cause of morbidity and mortality. Bedside, multimodal cerebral monitoring is a safe and promising technique for the diagnosis and prevention of secondary brain damage. The aim of this study is to investigate whether microdialysis parameters can be used as prognostic factors in patients with spontaneous cerebral bleeding, and their association with the long-term outcome.

## Methods

Twenty-seven patients with GCS <8 were included in the study. Mean age was 57.78 ± 9.94 years. The outcome of the patients was evaluated according to the Glasgow Outcome Scale (GOS) 3 and 6 months post-discharge. Data were evaluated using the SPSS 17.0 and *P *< 0.05.

## Results

In a linear statistical model that included all of the microdialysis parameters, only glucose was inversely associated with the patient outcome.

## Conclusions

We can use microdialysis to determine cerebral glucose levels, which we found to be associated with patient outcome.
